# Supplementary material for: Analyzing nested experimental designs—A user-friendly resampling method to determine experimental significance
Source: PLoS Comput Biol. 2022 May 2;18(5):e1010061. doi: 10.1371/journal.pcbi.1010061 (PMC9098003; doi:10.1371/journal.pcbi.1010061)
Supplement: S1 Text — (DOCX) [file pcbi.1010061.s001.docx]

**S1 Text**

The unbiased estimator for Var(Q) is as follows, where s_r,t_ is the cross power sum of X and Y:

$$S^{2}\left( Q \right)= \frac{1}{\left( -3+n \right)\left( -2+n \right)\left( -1+n \right)^{2}n^{2}} \left( \left( 6-4n \right)s_{0,1}^{2}s_{1,0}^{2}+\left( -n+n^{2} \right)s_{0,2}s_{1,0}^{2}+\left( -10n+6n^{2} \right)s_{0,1}s_{1,0}s_{1,1}+\left( 2n+n^{2}-n^{3} \right)s_{1,1}^{2}+\left( -2n+4n^{2}-2n^{3} \right)s_{1,0}s_{1,2}+\left( -n+n^{2} \right)s_{0,1}^{2}s_{2,0}+\left( n-n^{2} \right)s_{0,2}s_{2,0}+\left( -2n+4n^{2}-2n^{3} \right)s_{0,1}s_{2,1}+\left( n^{2}-2n^{3}+n^{4} \right)s_{2,2} \right)$$

$$s_{r,t}= \sum_{i=0}^{n} \left( X_{i} \right)^{r}\left( Y_{i} \right)^{t}$$
