# Supplementary figures and images for: Analyzing nested experimental designs—A user-friendly resampling method to determine experimental significance
Source: PLoS Comput Biol. 2022 May 2;18(5):e1010061. doi: 10.1371/journal.pcbi.1010061 (PMC9098003; doi:10.1371/journal.pcbi.1010061)

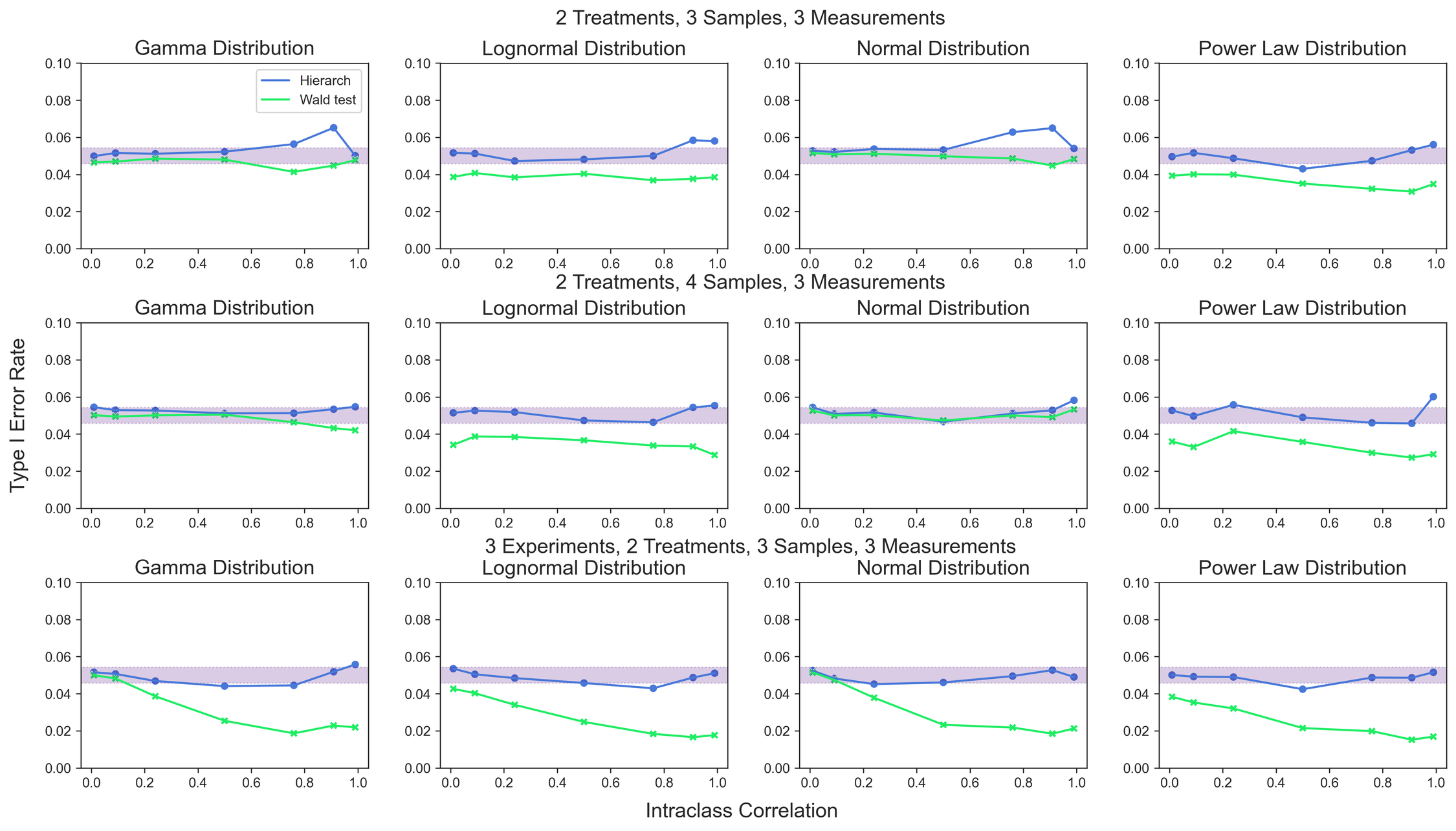

Supplement: S1 Fig — (TIF) [file pcbi.1010061.s002.tif]

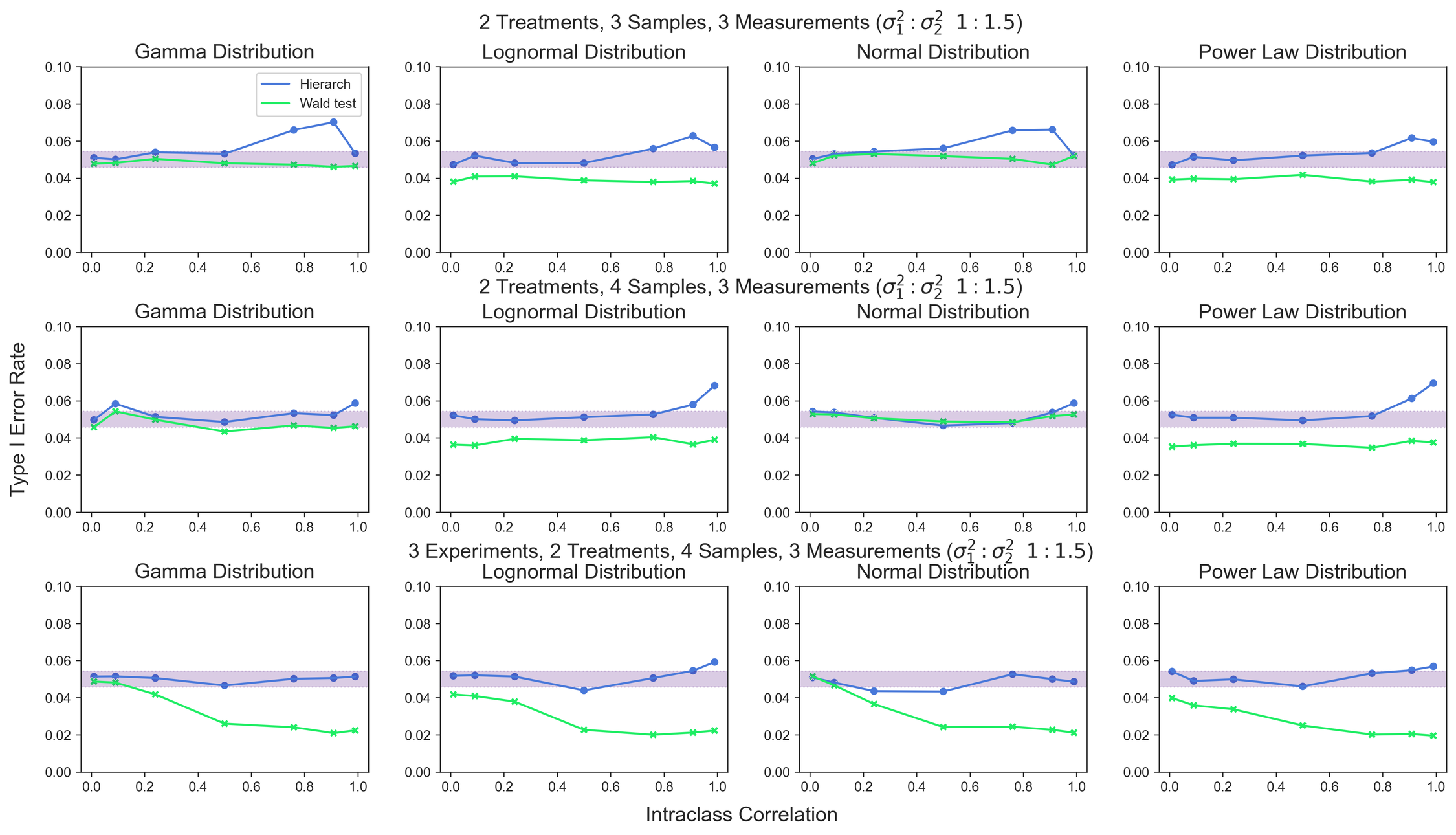

Supplement: S2 Fig — (TIF) [file pcbi.1010061.s003.tif]

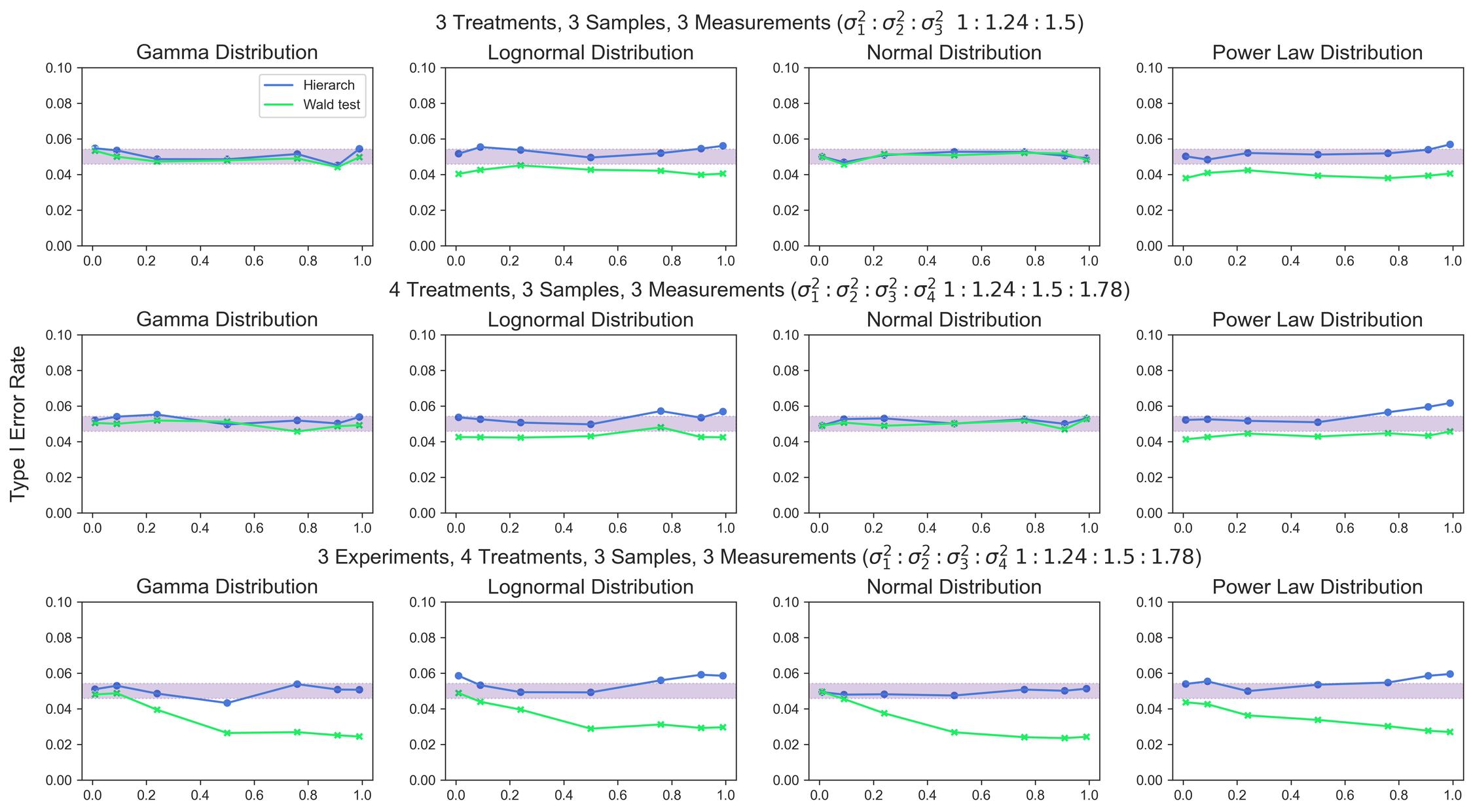

Supplement: S3 Fig — (TIF) [file pcbi.1010061.s004.tif]
