# Supplementary material for: Analyzing nested experimental designs—A user-friendly resampling method to determine experimental significance
Source: PLoS Comput Biol. 2022 May 2;18(5):e1010061. doi: 10.1371/journal.pcbi.1010061 (PMC9098003; doi:10.1371/journal.pcbi.1010061)
Supplement: Attachment — Submitted filename: Review of Analyzing Nested Experimental Designs.docx [file pcbi.1010061.s005.docx]

**Review of Analyzing Nested Experimental Designs - A User-Friendly Resampling Method to Determine Experimental Significance**

The authors have produced a solid pedagogical paper to accompany a very useful python package that facilitates the application of statistical tests to hierarchical datasets in the life sciences. As the authors have highlighted in their introduction, this has been a long running problem in the field with multiple proposed solutions. However, since datasets can vary widely between experiments as can scientific questions and hypotheses tested, no one has previously proposed a software package to take experimenter choice out of the equation. The paper introduces a software package backed up by rigorous statistical arguments and simulation studies to justify its use in a wide variety of experiments. However, I have some comments below that I believe will help strengthen the paper substantially by making it more accessible to a larger range of readers.

**Major Comments**

1. The content under the heading “Description of the hierarchical randomization test” is not clear. Given that this is the core of the proposed method, this section must both be expanded upon and made clearer. I would suggest building a toy example dataset (such as the one used in the early description and expanded upon in Example 2) and walking through the entire algorithm on that dataset. And while it is true that using the bootstrap makes minimal assumptions about the data generating process, decisions such as the number of samples to create in each bootstrapped sample introduce assumptions about the data generating process. This is alluded to under the “caveats” section but knowing default resampling parameters, as well as other available options, for the algorithm is an important consideration for experimenters seeking to use Hierarch.
2. An additional section detailing how the data needs to be formatted for Hierarch to perform statistical tests would be very useful. While this information can be obtained by parsing through the three examples or by looking up the documentation for the Hierarch package, a critical discussion of the assumptions Hierarch makes based on the structure of the dataframe and how it works would be valuable for future experimenters. Another point to clarify is how interaction effects work. Example 2 seems to suggest that simply repeating a column is sufficient to let Hierarch know to test the interaction effect. But a discussion of the assumptions here is missing. Can any column be duplicated? What happens if a column appears thrice? Also, the documentation for Hierarch mentions that interaction effects cannot be computed currently. Which interaction effects can and cannot currently be tested? Such questions are best addressed in a tutorial section on how to format one’s dataset prior to running Hierarch in the Methods section.
3. While I understand the rationale for the current structure, I believe the paper will be much easier to follow if the examples were under the Results section (part A) moving the contents of the current Results section to Results part B. Please explain all necessary background in the introduction and methods sections before diving into simulation results. And the code for the examples is not currently shared in the simulations repository. Please include this simulation code in the repository as well.
4. In the paper’s current form, the lack of testing on real experimental data stands out as a weakness. Even if the authors’ labs are dry labs lacking experimental data, there are now multiple datasets available online that the authors could test their package on and report if there are any major changes in interpretation between what was originally reported and their results. Demonstrating that their package has been applied successfully to real experimental data under multiple circumstances will provide confidence to future experimenters about the validity of adopting Hierarch.
5. While the authors discuss the limitations of applying linear mixed models to datasets with a small number of clusters as is typical in the life sciences, they do not make direct comparisons of the outputs of mixed models with that of Hierarch. Given that mixed models are currently considered the gold standard for analyzing hierarchical datasets, this is an important comparison to report.

**Minor Comments:**

1. Under “Construction of a studentized covariance test statistic”, equation numbers 5 through 7 are repeated. Please fix both the numbering and descriptions.
2. The switching back and forth between multiple terms to describe the various hierarchical levels is confusing to keep track of (biological and technical replicates versus experiments, treatments, samples and measurements). Please pick one term that can be applied across all datasets (for example, hierarchical level 1, 2 and so on and describe what that level is for each experiment / example).
3. Please always be consistent when reporting effect size and confidence interval measures. While most of the reported results use a 95% confidence interval, at the end of example 2, the authors switch to a 90% confidence interval. Please fix and check throughout.
