# Supplementary material for: Analyzing nested experimental designs—A user-friendly resampling method to determine experimental significance
Source: PLoS Comput Biol. 2022 May 2;18(5):e1010061. doi: 10.1371/journal.pcbi.1010061 (PMC9098003; doi:10.1371/journal.pcbi.1010061)
Supplement: Attachment — Submitted filename: Response to Reviewers PLOS Comp Bio.docx [file pcbi.1010061.s006.docx]

July 7, 2021

To Dr. Schneidman and the Editorial Board,

Please find our revision to **Analyzing Nested Experimental Designs – A User-Friendly Resampling Method to Determine Experimental Significance** attached. We have updated the manuscript in response to reviewer comments and replaced all of our figures with the PACE-corrected versions. Please find our response to the referee’s comments below, with the comments rendered in black and our response in red.

**Reviewer 1 Comments**

At the sample sizes suggested, the number of bootstrap samples at the lowest level of the hierarchy are quite limited, and so complete enumeration rather than resampling should be used. (e.g. with 3 images per well, there are only 27 possible bootstrap means, and even with 5 there are 5^5=3125). Clearly for examples where the bottom level of the hierarchy is large (such as Example 1) resampling will be required.

Yes, when full enumeration is possible, it should indeed be preferred. There are a number of common experimental designs that fully enumerating all of the options is well within the realm of possibility, which is why we included the option of specifying “exact” for the permutation test portion of Hierarch. The number of possible bootstrap samples when considering a nested design (6 wells with 27 possible bootstrap samples is already nearly 400 million resamples) quickly becomes difficult to fully enumerate, however, so we neglected to provide that option there. We have added a comment to this effect in the paragraph explaining the hierarchical randomization test:

“The bootstrap procedure involves resampling the within-cluster observations with replacement and recomputing the mean many times (say, 1000, as there are now 27^6^ possible combinations of bootstrapped wells), resulting in a distribution of means that, importantly, reflect the standard error and skew of the original observations.”

The authors have demonstrated that the test statistics holds its Type 1 error rate under various scenarios and that the derived confidence intervals have the required coverage. However, they do not address power. The high coverage of the intervals for the parametric tests seems to indicate that the parametric intervals are too wide and this should be stated somewhere.

This is a point that bears mentioning. Indeed, the over-coverage of the parametric tests results in lower power as compared to hierarchical randomization. We have added two paragraphs to the Simulation Results section discussing this.

I think that referring to the bootstrap distribution as the “full posterior” is confusing. It is not the posterior – it is an estimate of the population distribution.

We thank the referee for this perspective. With that statement, we were alluding to the observation made in Rubin, 1981 that the difference between Efron bootstrap and the Bayesian bootstrap are primarily differences in intuition. However, we agree that this statement may be distracting from the overall point, so we have adjusted that sentence to the following:

“In this manner, hierarchical randomization tests have an element of a Bayesian approach - rather than summarizing information from lower levels using point estimates, the randomization test is performed on the full sampling distribution of each cluster.”

I would suggest not comparing the randomization test to the jackknife as the intended audience likely is not familiar with the latter.

This is a good point. We’ve adjusted the phrasing of that sentence to the following:

“Hierarchical randomization hypothesis tests are versatile – they can be applied to a wide variety of experimental designs while maintaining better control of Type I error rates than asymptotic tests.”

This is a very quotable statement. Perhaps it could be highlighted in some way. “…the strong assumptions of an asymptotic test which … are doing at least as much work as the data”

We agree that the parenthetical makes it easier to gloss over this sentence, so we have changed it to the following:

“Ordinarily, this leaves the researcher stuck between a rock and a hard place - either they have to go with the strong assumptions of an asymptotic test (which are doing at least as much work as the data is) or accept that they cannot achieve p < 0.05 with a nonparametric test.”

Equation 7 appears to be the sample crossed moment, rather than the population crossed moment.

Equation 7 is the maximum likelihood estimator for any population cross moment. In the case of covariance, it is missing Bessel’s correction, so it is not the sample crossed moment.

The code uses the order of the data columns to determine the hierarchy. It might be better to have a command line that gives the order, which would also document the hierarchy explicitly.

We agree with this recommendation. Our goal is to implement a Wilkinson-like notation for specifying experimental designs – i.e. “Fluorescence ~ (Mice + Treatment) | Wells.” Unfortunately, there are no off-the-shelf implementations of Wilkinson notation in Python that we are aware of that we can readily adapt to Hierarch, so we are working on extending one of them for our purposes. Ideally, this will be in Hierarch version 2.0.

Is it possible to have crossed factors? E.g. Perhaps at the top level (mice) males and females, treated and untreated or a split plot design, such as treated and untreated mice and wells with different drugs? It is not clear to me if the code can handle this, and if so, how it would be indicated.

Yes, though a current limitation is that tests for crossed factors must be set up manually. As pointed out in Anderson and Braak, 2003, exact tests for crossed designs can be constructed by treating the factor under consideration as nested within any factors it is crossed with. As pointed out in the above comment, Hierarch does not yet implement a strategy to directly specify whether two columns in a design matrix represent two different levels or are merely crossed factors on the same levels. We chose to handle this in Hierarch v1 by assuming different columns are different levels and relying on the users to manually nest their factor of interest themselves. However, this is error-prone, so we hope to solve this with the strategy detailed in the comment above. In the meantime, we have added a discussion about crossed factors in the Methods section.

**Reviewer 2 Comments**

**Major Comments**

1. The content under the heading “Description of the hierarchical randomization test” is not clear. Given that this is the core of the proposed method, this section must both be expanded upon and made clearer. I would suggest building a toy example dataset (such as the one used in the early description and expanded upon in Example 2) and walking through the entire algorithm on that dataset. And while it is true that using the bootstrap makes minimal assumptions about the data generating process, decisions such as the number of samples to create in each bootstrapped sample introduce assumptions about the data generating process. This is alluded to under the “caveats” section but knowing default resampling parameters, as well as other available options, for the algorithm is an important consideration for experimenters seeking to use Hierarch.

We thank the referee for this suggestion – indeed, upon review, we agree that this section is a little unclear. We have expanded it significantly by referring explicitly to the experiment in Scheme 2 and broken up the steps into smaller logical units. We also have added commentary about what the default values for the number of permutations and bootstraps are.

2. An additional section detailing how the data needs to be formatted for Hierarch to perform statistical tests would be very useful. While this information can be obtained by parsing through the three examples or by looking up the documentation for the Hierarch package, a critical discussion of the assumptions Hierarch makes based on the structure of the dataframe and how it works would be valuable for future experimenters. Another point to clarify is how interaction effects work. Example 2 seems to suggest that simply repeating a column is sufficient to let Hierarch know to test the interaction effect. But a discussion of the assumptions here is missing. Can any column be duplicated? What happens if a column appears thrice? Also, the documentation for Hierarch mentions that interaction effects cannot be computed currently. Which interaction effects can and cannot currently be tested? Such questions are best addressed in a tutorial section on how to format one’s dataset prior to running Hierarch in the Methods section.

We agree that adding a discussion about data formatting before diving into the examples and simulation results will help frame the later discussions. Furthermore, it will allow users to follow the manuscript as a guide to using *Hierarch* to analyze their data. We have moved the Methods section to immediately after the Introduction and expanded it significantly by adding subsections about Data Formatting, Crossed Factors, and Interaction Effects.

3. While I understand the rationale for the current structure, I believe the paper will be much easier to follow if the examples were under the Results section (part A) moving the contents of the current Results section to Results part B. Please explain all necessary background in the introduction and methods sections before diving into simulation results. And the code for the examples is not currently shared in the simulations repository. Please include this simulation code in the repository as well.

To address this and the previous suggestion, we have moved the Methods section to before the examples and expanded it to contain the description of the algorithm and the studentized covariance test statistic. We have also uploaded the code producing the examples and figures to the hierarch-simulations repository.

4. In the paper’s current form, the lack of testing on real experimental data stands out as a weakness. Even if the authors’ labs are dry labs lacking experimental data, there are now multiple datasets available online that the authors could test their package on and report if there are any major changes in interpretation between what was originally reported and their results. Demonstrating that their package has been applied successfully to real experimental data under multiple circumstances will provide confidence to future experimenters about the validity of adopting Hierarch.

We thank the referee for this suggestion. We considered including real experimental data, as many statistical methods papers include real experimental data. However, we instead chose to provide examples from simulated data (though, the simulated data is meant to mimic experiments performed in our lab).

We reasoned that the validity of a novel frequentist method (such as a new hypothesis test) depends on the demonstration of its long-run properties, such as Type I error rate control, over many analyses. Unfortunately, it is difficult to produce 10,000 datasets for a given experimental design and even if we could, we would still have no knowledge of the true parameters of the data-generating process (though we would hope to have a good estimate at that point!). As a result, we decided that adding a section on real experimental data would not add much clarity beyond that added by the Examples and Simulations sections.

5. While the authors discuss the limitations of applying linear mixed models to datasets with a small number of clusters as is typical in the life sciences, they do not make direct comparisons of the outputs of mixed models with that of Hierarch. Given that mixed models are currently considered the gold standard for analyzing hierarchical datasets, this is an important comparison to report.

This is an excellent suggestion. We have added this comparison in Figure 11, as well as a discussion of unbiased and biased estimation.

**Minor Comments:**

1. Under “Construction of a studentized covariance test statistic”, equation numbers 5 through 7 are repeated. Please fix both the numbering and descriptions.

We have harmonized the numbering and descriptions.

2. The switching back and forth between multiple terms to describe the various hierarchical levels is confusing to keep track of (biological and technical replicates versus experiments, treatments, samples and measurements). Please pick one term that can be applied across all datasets (for example, hierarchical level 1, 2 and so on and describe what that level is for each experiment / example).

Thank you for this suggestion – we agree that it helps improve clarity. Every formula has been rewritten with a consistent nomenclature (Level 1, Level 2, etc.) and captions beneath each equation now describe how those levels relate to the dataset at hand.

3. Please always be consistent when reporting effect size and confidence interval measures. While most of the reported results use a 95% confidence interval, at the end of example 2, the authors switch to a 90% confidence interval. Please fix and check throughout.

Thank you for your keen eye. Upon review, we note that due to the small number of possible permutations when considering the interaction effect, describing an asymptotically exact 95% confidence interval becomes impossible. We changed the text of the paragraph to the following to point this out:

“Accounting for treatment effect heterogeneity reduces the possible number of permutations (from 20^3^ = 4000 to 2^3^ = 8). This increases the *p-*value (0.118) widens the confidence interval (0.478, 23.45, 90% confidence interval). We note that because of the severely restricted number of permutations, it is not possible to compute a 95% confidence interval for the main effect – the smallest tail probability that *Hierarch* can reliably describe is inversely proportional to twice the number of possible permutations.”

All authors have read the updated manuscript and consented to publication of these results.

Thank you for your consideration,

Carolyn Bertozzi
